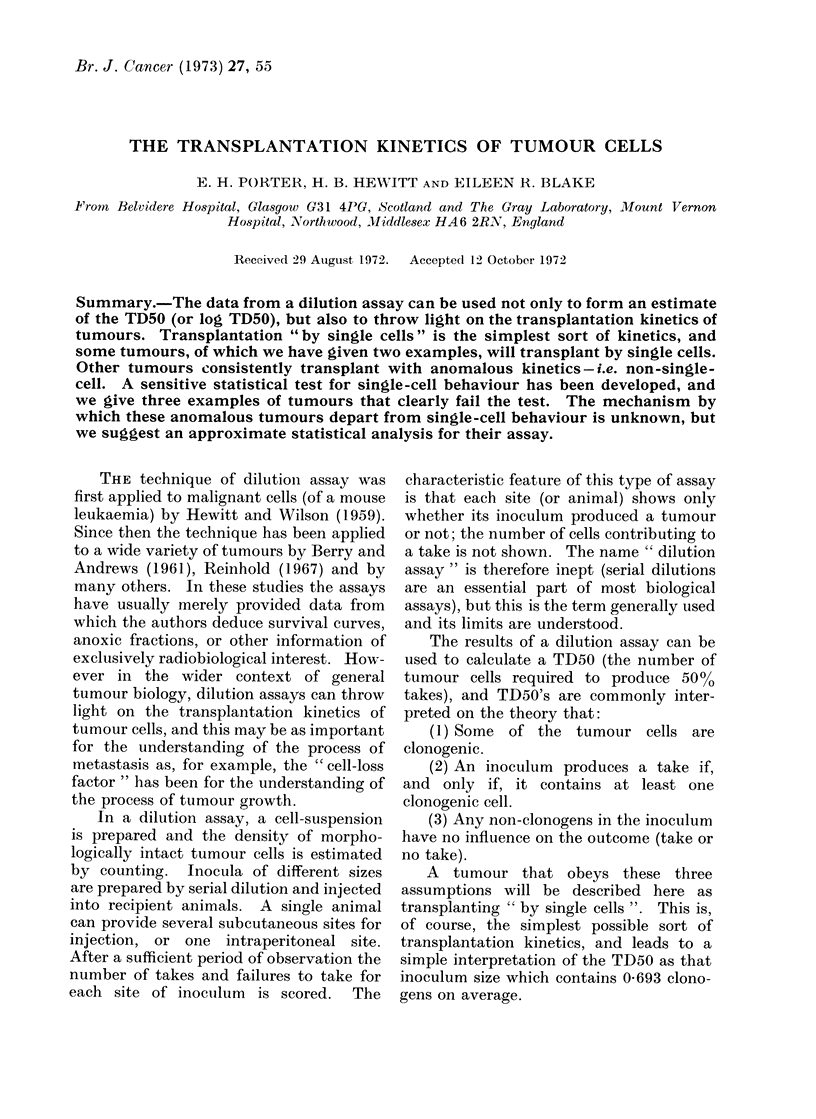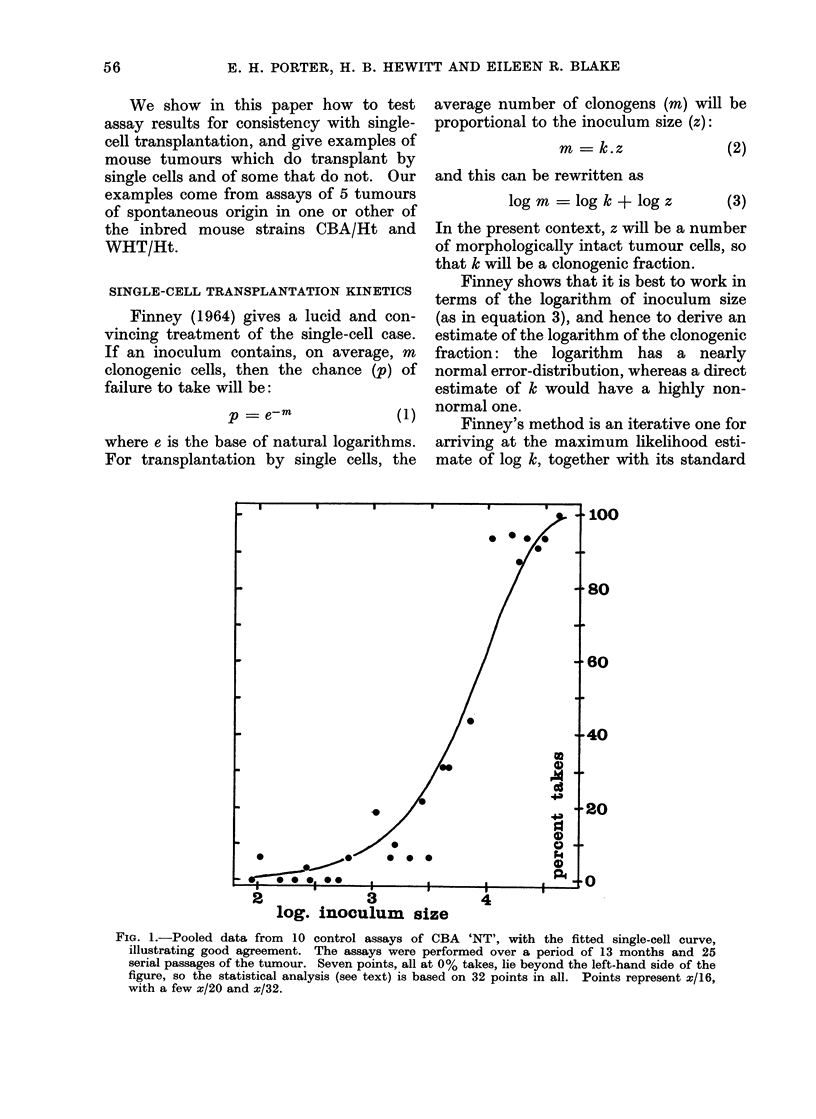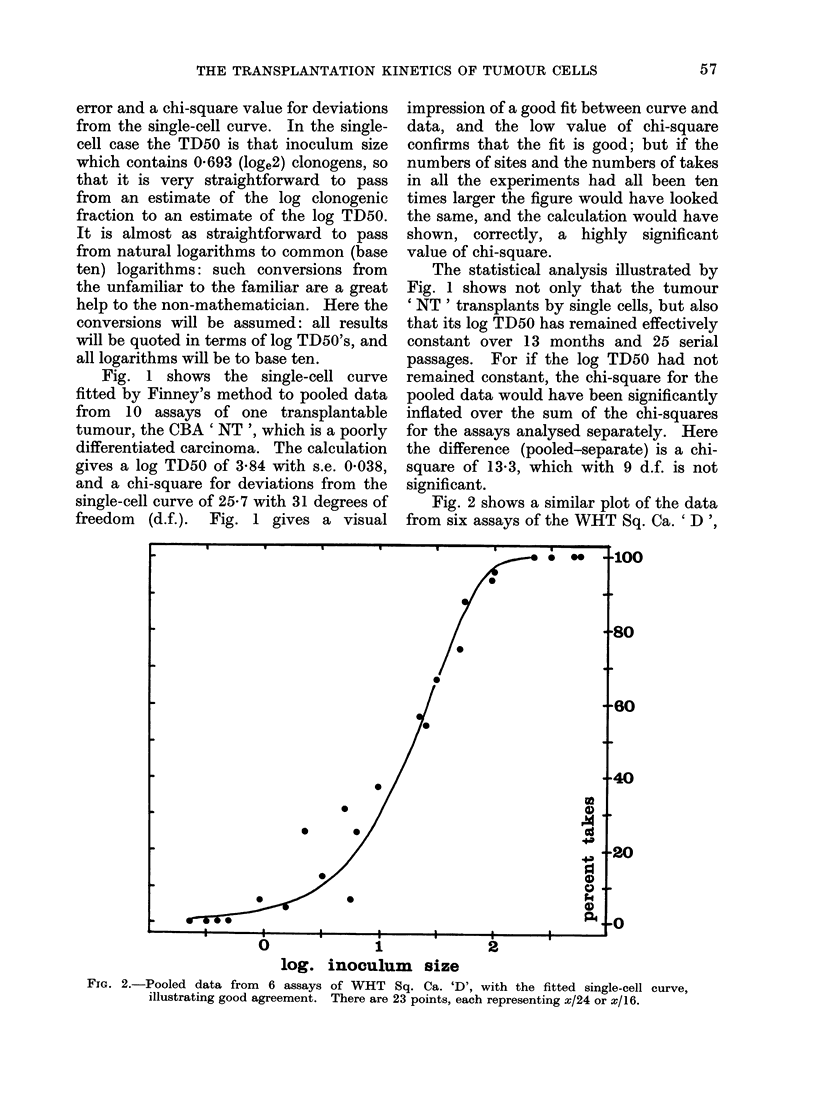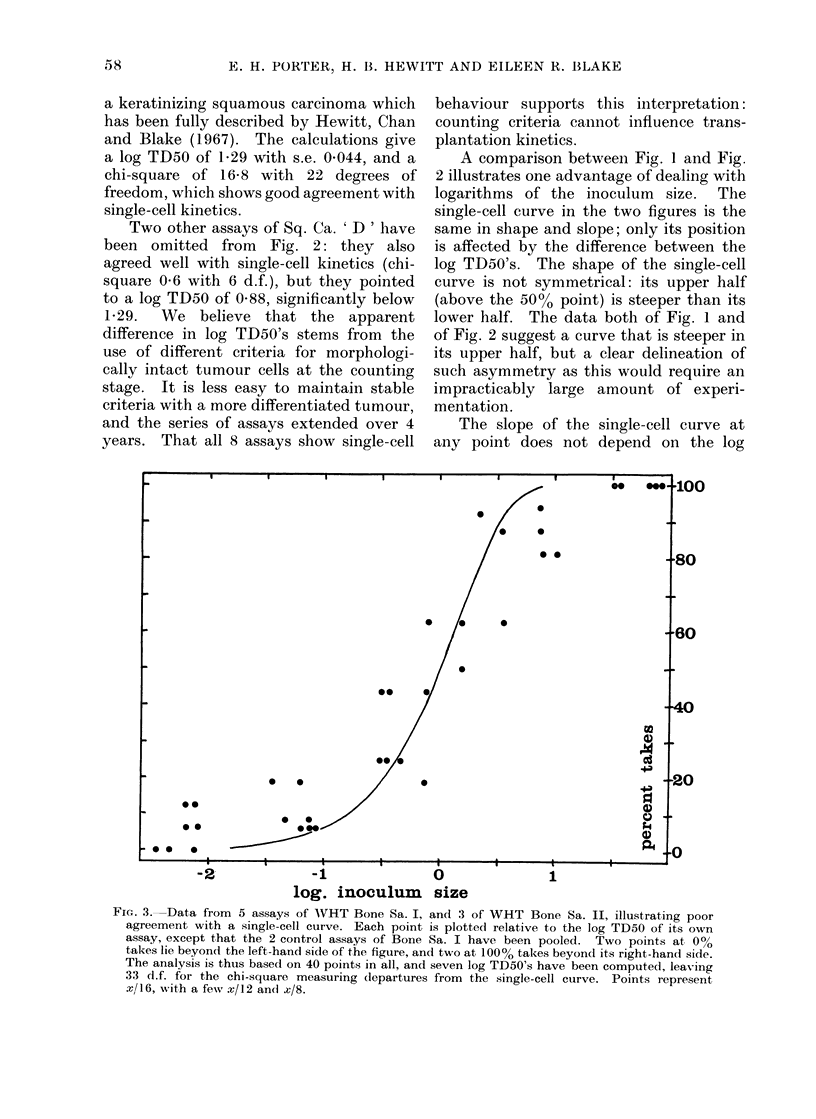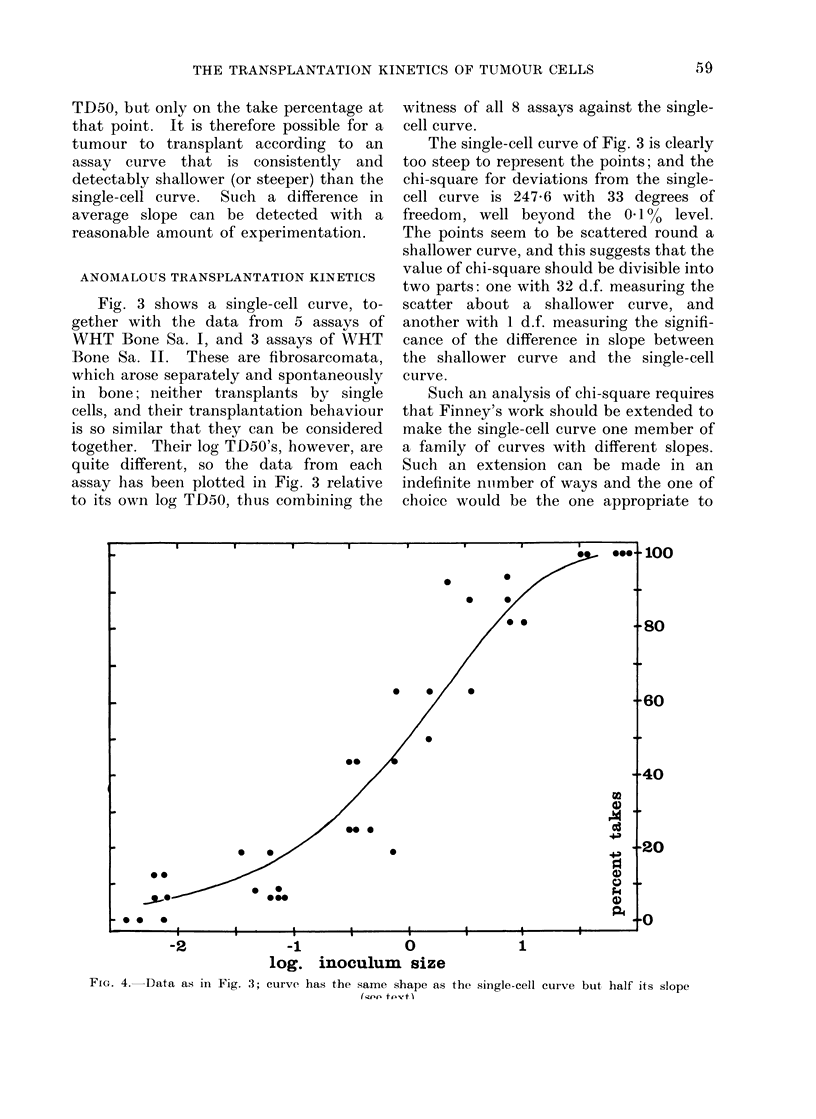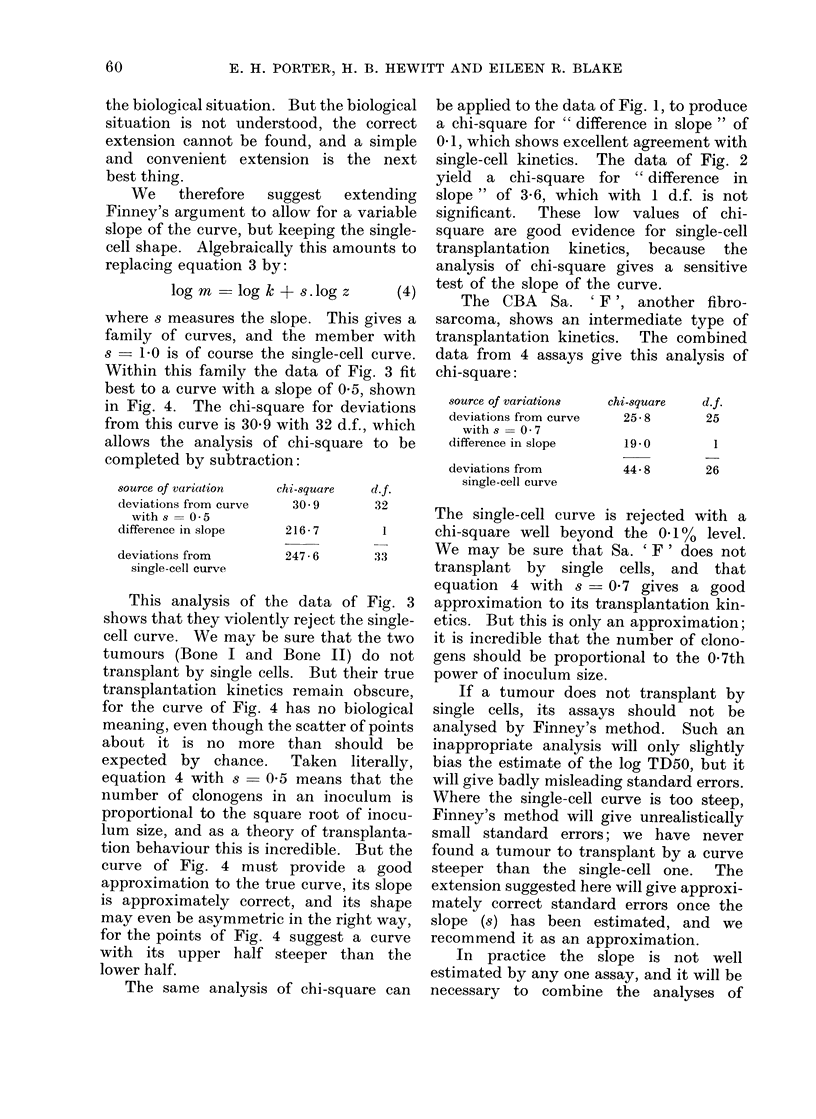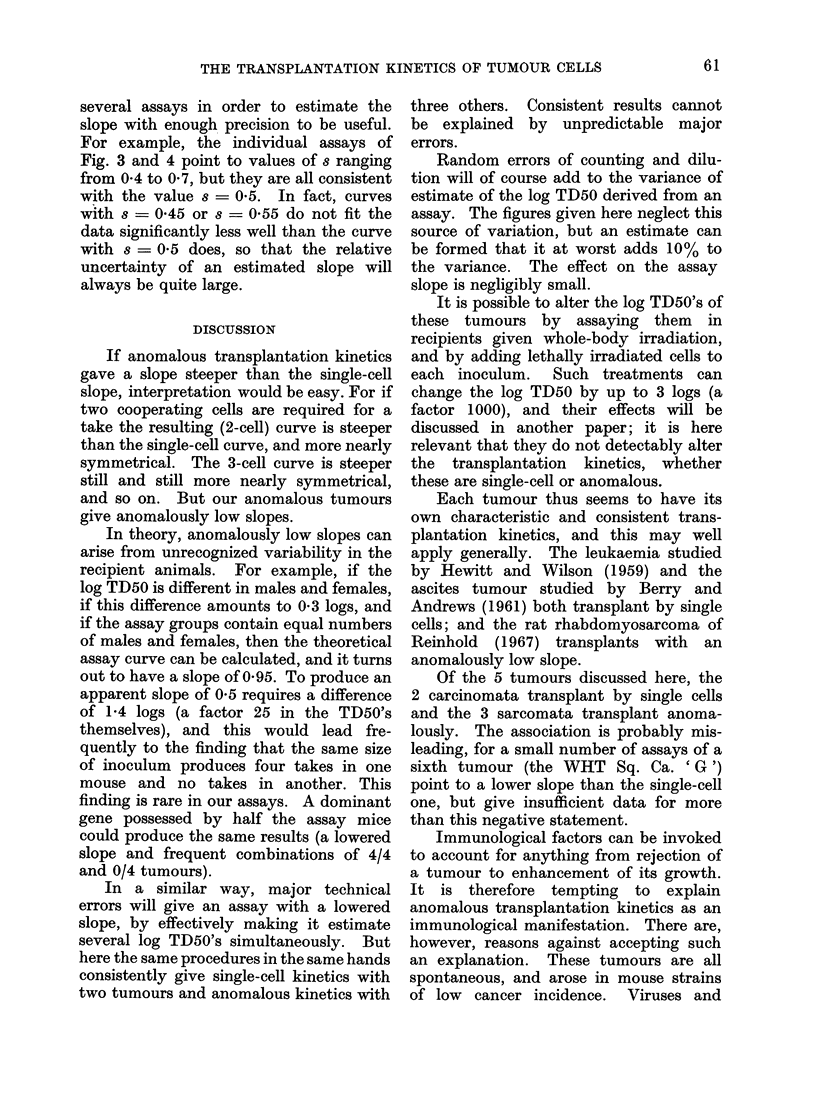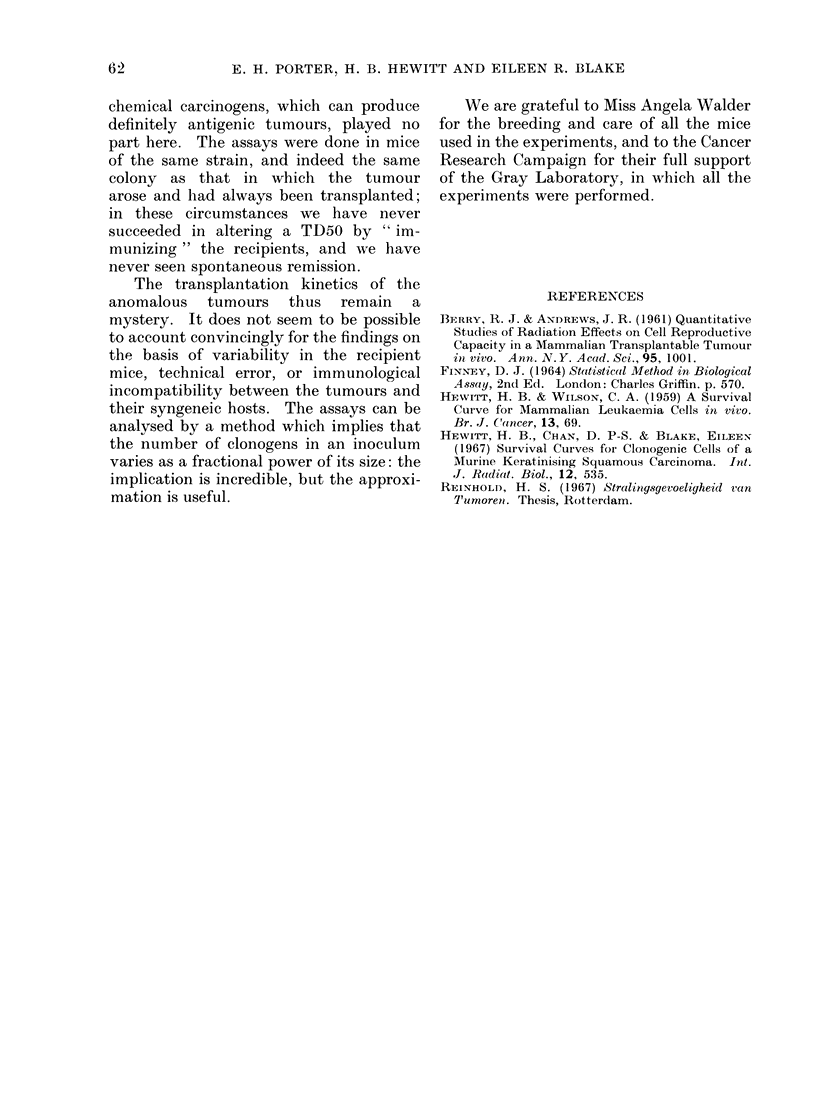# The Transplantation Kinetics of Tumour Cells

**DOI:** 10.1038/bjc.1973.7

**Published:** 1973-01

**Authors:** E. H. Porter, H. B. Hewitt, Eileen R. Blake

## Abstract

The data from a dilution assay can be used not only to form an estimate of the TD50 (or log TD50), but also to throw light on the transplantation kinetics of tumours. Transplantation “by single cells” is the simplest sort of kinetics, and some tumours, of which we have given two examples, will transplant by single cells. Other tumours consistently transplant with anomalous kinetics-*i.e.* non-single-cell. A sensitive statistical test for single-cell behaviour has been developed, and we give three examples of tumours that clearly fail the test. The mechanism by which these anomalous tumours depart from single-cell behaviour is unknown, but we suggest an approximate statistical analysis for their assay.


*Br. J. Cancer* (1973) **27**, 55

### THE TRANSPLANTATION KINETICS OF TUMOUR CELLS

E. H. PORTER, H. B. HEWITT AND EILEEN R. BLAKE

*From Belvidere Hospital, Glasgow G31 4PG, Scotland and The Gray Laboratory, Mount Vernon Hospital, Northwood, Middlesex HA6 2RN, England*

Received 29 August 1972. Accepted 12 October 1972

**Summary.—The data from a dilution assay can be used not only to form an estimate of the TD50 (or log TD50), but also to throw light on the transplantation kinetics of tumours. Transplantation "by single cells" is the simplest sort of kinetics, and some tumours, of which we have given two examples, will transplant by single cells. Other tumours consistently transplant with anomalous kinetics—*i.e.* non-single-cell. A sensitive statistical test for single-cell behaviour has been developed, and we give three examples of tumours that clearly fail the test. The mechanism by which these anomalous tumours depart from single-cell behaviour is unknown, but we suggest an approximate statistical analysis for their assay.**

THE technique of dilution assay was first applied to malignant cells (of a mouse leukaemia) by Hewitt and Wilson (1959). Since then the technique has been applied to a wide variety of tumours by Berry and Andrews (1961), Reinhold (1967) and by many others. In these studies the assays have usually merely provided data from which the authors deduce survival curves, anoxic fractions, or other information of exclusively radiobiological interest. However in the wider context of general tumour biology, dilution assays can throw light on the transplantation kinetics of tumour cells, and this may be as important for the understanding of the process of metastasis as, for example, the "cell-loss factor" has been for the understanding of the process of tumour growth.

In a dilution assay, a cell-suspension is prepared and the density of morphologically intact tumour cells is estimated by counting. Inocula of different sizes are prepared by serial dilution and injected into recipient animals. A single animal can provide several subcutaneous sites for injection, or one intraperitoneal site. After a sufficient period of observation the number of takes and failures to take for each site of inoculum is scored. The characteristic feature of this type of assay is that each site (or animal) shows only whether its inoculum produced a tumour or not; the number of cells contributing to a take is not shown. The name "dilution assay" is therefore inept (serial dilutions are an essential part of most biological assays), but this is the term generally used and its limits are understood.

The results of a dilution assay can be used to calculate a TD50 (the number of tumour cells required to produce 50% takes), and TD50's are commonly interpreted on the theory that:

(1) Some of the tumour cells are clonogenic.

(2) An inoculum produces a take if, and only if, it contains at least one clonogenic cell.

(3) Any non-clonogens in the inoculum have no influence on the outcome (take or no take).

A tumour that obeys these three assumptions will be described here as transplanting "by single cells". This is, of course, the simplest possible sort of transplantation kinetics, and leads to a simple interpretation of the TD50 as that inoculum size which contains 0·693 clonogens on average.

We show in this paper how to test assay results for consistency with single-cell transplantation, and give examples of mouse tumours which do transplant by single cells and of some that do not. Our examples come from assays of 5 tumours of spontaneous origin in one or other of the inbred mouse strains CBA/Ht and WHT/Ht.

### SINGLE-CELL TRANSPLANTATION KINETICS

Finney (1964) gives a lucid and convincing treatment of the single-cell case. If an inoculum contains, on average, $m$ clonogenic cells, then the chance ($p$) of failure to take will be:

$$p = e^{-m} \quad (1)$$

where $e$ is the base of natural logarithms. For transplantation by single cells, the average number of clonogens ($m$) will be proportional to the inoculum size ($z$):

$$m = k.z \quad (2)$$

and this can be rewritten as

$$\log m = \log k + \log z \quad (3)$$

In the present context, $z$ will be a number of morphologically intact tumour cells, so that $k$ will be a clonogenic fraction.

Finney shows that it is best to work in terms of the logarithm of inoculum size (as in equation 3), and hence to derive an estimate of the logarithm of the clonogenic fraction: the logarithm has a nearly normal error-distribution, whereas a direct estimate of $k$ would have a highly non-normal one.

Finney's method is an iterative one for arriving at the maximum likelihood estimate of log $k$, together with its standard

FIG. 1.—Pooled data from 10 control assays of CBA 'NT', with the fitted single-cell curve, illustrating good agreement. The assays were performed over a period of 13 months and 25 serial passages of the tumour. Seven points, all at 0% takes, lie beyond the left-hand side of the figure, so the statistical analysis (see text) is based on 32 points in all. Points represent $x/16$, with a few $x/20$ and $x/32$.

error and a chi-square value for deviations from the single-cell curve. In the single-cell case the TD50 is that inoculum size which contains 0·693 ($\log_e 2$) clonogens, so that it is very straightforward to pass from an estimate of the log clonogenic fraction to an estimate of the log TD50. It is almost as straightforward to pass from natural logarithms to common (base ten) logarithms: such conversions from the unfamiliar to the familiar are a great help to the non-mathematician. Here the conversions will be assumed: all results will be quoted in terms of log TD50's, and all logarithms will be to base ten.

Fig. 1 shows the single-cell curve fitted by Finney's method to pooled data from 10 assays of one transplantable tumour, the CBA ' NT ', which is a poorly differentiated carcinoma. The calculation gives a log TD50 of 3·84 with s.e. 0·038, and a chi-square for deviations from the single-cell curve of 25·7 with 31 degrees of freedom (d.f.). Fig. 1 gives a visual impression of a good fit between curve and data, and the low value of chi-square confirms that the fit is good; but if the numbers of sites and the numbers of takes in all the experiments had all been ten times larger the figure would have looked the same, and the calculation would have shown, correctly, a highly significant value of chi-square.

The statistical analysis illustrated by Fig. 1 shows not only that the tumour ' NT ' transplants by single cells, but also that its log TD50 has remained effectively constant over 13 months and 25 serial passages. For if the log TD50 had not remained constant, the chi-square for the pooled data would have been significantly inflated over the sum of the chi-squares for the assays analysed separately. Here the difference (pooled–separate) is a chi-square of 13·3, which with 9 d.f. is not significant.

Fig. 2 shows a similar plot of the data from six assays of the WHT Sq. Ca. ' D ',

FIG. 2.—Pooled data from 6 assays of WHT Sq. Ca. 'D', with the fitted single-cell curve, illustrating good agreement. There are 23 points, each representing $x/24$ or $x/16$.

a keratinizing squamous carcinoma which has been fully described by Hewitt, Chan and Blake (1967). The calculations give a log TD50 of 1·29 with s.e. 0·044, and a chi-square of 16·8 with 22 degrees of freedom, which shows good agreement with single-cell kinetics.

Two other assays of Sq. Ca. ' D ' have been omitted from Fig. 2: they also agreed well with single-cell kinetics (chi-square 0·6 with 6 d.f.), but they pointed to a log TD50 of 0·88, significantly below 1·29. We believe that the apparent difference in log TD50's stems from the use of different criteria for morphologically intact tumour cells at the counting stage. It is less easy to maintain stable criteria with a more differentiated tumour, and the series of assays extended over 4 years. That all 8 assays show single-cell behaviour supports this interpretation: counting criteria cannot influence transplantation kinetics.

A comparison between Fig. 1 and Fig. 2 illustrates one advantage of dealing with logarithms of the inoculum size. The single-cell curve in the two figures is the same in shape and slope; only its position is affected by the difference between the log TD50's. The shape of the single-cell curve is not symmetrical: its upper half (above the 50% point) is steeper than its lower half. The data both of Fig. 1 and of Fig. 2 suggest a curve that is steeper in its upper half, but a clear delineation of such asymmetry as this would require an impracticably large amount of experimentation.

The slope of the single-cell curve at any point does not depend on the log

FIG. 3.—Data from 5 assays of WHT Bone Sa. I, and 3 of WHT Bone Sa. II, illustrating poor agreement with a single-cell curve. Each point is plotted relative to the log TD50 of its own assay, except that the 2 control assays of Bone Sa. I have been pooled. Two points at 0% takes lie beyond the left-hand side of the figure, and two at 100% takes beyond its right-hand side. The analysis is thus based on 40 points in all, and seven log TD50's have been computed, leaving 33 d.f. for the chi-square measuring departures from the single-cell curve. Points represent $x/16$, with a few $x/12$ and $x/8$.

TD50, but only on the take percentage at that point. It is therefore possible for a tumour to transplant according to an assay curve that is consistently and detectably shallower (or steeper) than the single-cell curve. Such a difference in average slope can be detected with a reasonable amount of experimentation.

### ANOMALOUS TRANSPLANTATION KINETICS

Fig. 3 shows a single-cell curve, together with the data from 5 assays of WHT Bone Sa. I, and 3 assays of WHT Bone Sa. II. These are fibrosarcomata, which arose separately and spontaneously in bone; neither transplants by single cells, and their transplantation behaviour is so similar that they can be considered together. Their log TD50's, however, are quite different, so the data from each assay has been plotted in Fig. 3 relative to its own log TD50, thus combining the witness of all 8 assays against the single-cell curve.

The single-cell curve of Fig. 3 is clearly too steep to represent the points; and the chi-square for deviations from the single-cell curve is 247·6 with 33 degrees of freedom, well beyond the 0·1% level. The points seem to be scattered round a shallower curve, and this suggests that the value of chi-square should be divisible into two parts: one with 32 d.f. measuring the scatter about a shallower curve, and another with 1 d.f. measuring the significance of the difference in slope between the shallower curve and the single-cell curve.

Such an analysis of chi-square requires that Finney's work should be extended to make the single-cell curve one member of a family of curves with different slopes. Such an extension can be made in an indefinite number of ways and the one of choice would be the one appropriate to

FIG. 4.—Data as in Fig. 3; curve has the same shape as the single-cell curve but half its slope (see text).

the biological situation. But the biological situation is not understood, the correct extension cannot be found, and a simple and convenient extension is the next best thing.

We therefore suggest extending Finney's argument to allow for a variable slope of the curve, but keeping the single-cell shape. Algebraically this amounts to replacing equation 3 by:

$$\log m = \log k + s.\log z \qquad (4)$$

where $s$ measures the slope. This gives a family of curves, and the member with $s = 1{\cdot}0$ is of course the single-cell curve. Within this family the data of Fig. 3 fit best to a curve with a slope of 0·5, shown in Fig. 4. The chi-square for deviations from this curve is 30·9 with 32 d.f., which allows the analysis of chi-square to be completed by subtraction:

| *source of variation* | *chi-square* | *d.f.* |
|---|---|---|
| deviations from curve with $s = 0{\cdot}5$ | 30·9 | 32 |
| difference in slope | 216·7 | 1 |
| deviations from single-cell curve | 247·6 | 33 |

This analysis of the data of Fig. 3 shows that they violently reject the single-cell curve. We may be sure that the two tumours (Bone I and Bone II) do not transplant by single cells. But their true transplantation kinetics remain obscure, for the curve of Fig. 4 has no biological meaning, even though the scatter of points about it is no more than should be expected by chance. Taken literally, equation 4 with $s = 0{\cdot}5$ means that the number of clonogens in an inoculum is proportional to the square root of inoculum size, and as a theory of transplantation behaviour this is incredible. But the curve of Fig. 4 must provide a good approximation to the true curve, its slope is approximately correct, and its shape may even be asymmetric in the right way, for the points of Fig. 4 suggest a curve with its upper half steeper than the lower half.

The same analysis of chi-square can be applied to the data of Fig. 1, to produce a chi-square for " difference in slope " of 0·1, which shows excellent agreement with single-cell kinetics. The data of Fig. 2 yield a chi-square for " difference in slope " of 3·6, which with 1 d.f. is not significant. These low values of chi-square are good evidence for single-cell transplantation kinetics, because the analysis of chi-square gives a sensitive test of the slope of the curve.

The CBA Sa. ' F ', another fibrosarcoma, shows an intermediate type of transplantation kinetics. The combined data from 4 assays give this analysis of chi-square:

| *source of variations* | *chi-square* | *d.f.* |
|---|---|---|
| deviations from curve with $s = 0{\cdot}7$ | 25·8 | 25 |
| difference in slope | 19·0 | 1 |
| deviations from single-cell curve | 44·8 | 26 |

The single-cell curve is rejected with a chi-square well beyond the 0·1% level. We may be sure that Sa. ' F ' does not transplant by single cells, and that equation 4 with $s = 0{\cdot}7$ gives a good approximation to its transplantation kinetics. But this is only an approximation; it is incredible that the number of clonogens should be proportional to the 0·7th power of inoculum size.

If a tumour does not transplant by single cells, its assays should not be analysed by Finney's method. Such an inappropriate analysis will only slightly bias the estimate of the log TD50, but it will give badly misleading standard errors. Where the single-cell curve is too steep, Finney's method will give unrealistically small standard errors; we have never found a tumour to transplant by a curve steeper than the single-cell one. The extension suggested here will give approximately correct standard errors once the slope ($s$) has been estimated, and we recommend it as an approximation.

In practice the slope is not well estimated by any one assay, and it will be necessary to combine the analyses of several assays in order to estimate the slope with enough precision to be useful. For example, the individual assays of Fig. 3 and 4 point to values of $s$ ranging from 0·4 to 0·7, but they are all consistent with the value $s = 0{\cdot}5$. In fact, curves with $s = 0{\cdot}45$ or $s = 0{\cdot}55$ do not fit the data significantly less well than the curve with $s = 0{\cdot}5$ does, so that the relative uncertainty of an estimated slope will always be quite large.

### DISCUSSION

If anomalous transplantation kinetics gave a slope steeper than the single-cell slope, interpretation would be easy. For if two cooperating cells are required for a take the resulting (2-cell) curve is steeper than the single-cell curve, and more nearly symmetrical. The 3-cell curve is steeper still and still more nearly symmetrical, and so on. But our anomalous tumours give anomalously low slopes.

In theory, anomalously low slopes can arise from unrecognized variability in the recipient animals. For example, if the log TD50 is different in males and females, if this difference amounts to 0·3 logs, and if the assay groups contain equal numbers of males and females, then the theoretical assay curve can be calculated, and it turns out to have a slope of 0·95. To produce an apparent slope of 0·5 requires a difference of 1·4 logs (a factor 25 in the TD50's themselves), and this would lead frequently to the finding that the same size of inoculum produces four takes in one mouse and no takes in another. This finding is rare in our assays. A dominant gene possessed by half the assay mice could produce the same results (a lowered slope and frequent combinations of 4/4 and 0/4 tumours).

In a similar way, major technical errors will give an assay with a lowered slope, by effectively making it estimate several log TD50's simultaneously. But here the same procedures in the same hands consistently give single-cell kinetics with two tumours and anomalous kinetics with three others. Consistent results cannot be explained by unpredictable major errors.

Random errors of counting and dilution will of course add to the variance of estimate of the log TD50 derived from an assay. The figures given here neglect this source of variation, but an estimate can be formed that it at worst adds 10% to the variance. The effect on the assay slope is negligibly small.

It is possible to alter the log TD50's of these tumours by assaying them in recipients given whole-body irradiation, and by adding lethally irradiated cells to each inoculum. Such treatments can change the log TD50 by up to 3 logs (a factor 1000), and their effects will be discussed in another paper; it is here relevant that they do not detectably alter the transplantation kinetics, whether these are single-cell or anomalous.

Each tumour thus seems to have its own characteristic and consistent transplantation kinetics, and this may well apply generally. The leukaemia studied by Hewitt and Wilson (1959) and the ascites tumour studied by Berry and Andrews (1961) both transplant by single cells; and the rat rhabdomyosarcoma of Reinhold (1967) transplants with an anomalously low slope.

Of the 5 tumours discussed here, the 2 carcinomata transplant by single cells and the 3 sarcomata transplant anomalously. The association is probably misleading, for a small number of assays of a sixth tumour (the WHT Sq. Ca. 'G') point to a lower slope than the single-cell one, but give insufficient data for more than this negative statement.

Immunological factors can be invoked to account for anything from rejection of a tumour to enhancement of its growth. It is therefore tempting to explain anomalous transplantation kinetics as an immunological manifestation. There are, however, reasons against accepting such an explanation. These tumours are all spontaneous, and arose in mouse strains of low cancer incidence. Viruses and chemical carcinogens, which can produce definitely antigenic tumours, played no part here. The assays were done in mice of the same strain, and indeed the same colony as that in which the tumour arose and had always been transplanted; in these circumstances we have never succeeded in altering a TD50 by "immunizing" the recipients, and we have never seen spontaneous remission.

The transplantation kinetics of the anomalous tumours thus remain a mystery. It does not seem to be possible to account convincingly for the findings on the basis of variability in the recipient mice, technical error, or immunological incompatibility between the tumours and their syngeneic hosts. The assays can be analysed by a method which implies that the number of clonogens in an inoculum varies as a fractional power of its size: the implication is incredible, but the approximation is useful.

We are grateful to Miss Angela Walder for the breeding and care of all the mice used in the experiments, and to the Cancer Research Campaign for their full support of the Gray Laboratory, in which all the experiments were performed.

REFERENCES

BERRY, R. J. & ANDREWS, J. R. (1961) Quantitative Studies of Radiation Effects on Cell Reproductive Capacity in a Mammalian Transplantable Tumour *in vivo*. *Ann. N.Y. Acad. Sci.*, **95**, 1001.

FINNEY, D. J. (1964) *Statistical Method in Biological Assay*, 2nd Ed. London: Charles Griffin. p. 570.

HEWITT, H. B. & WILSON, C. A. (1959) A Survival Curve for Mammalian Leukaemia Cells *in vivo*. *Br. J. Cancer*, **13**, 69.

HEWITT, H. B., CHAN, D. P-S. & BLAKE, EILEEN (1967) Survival Curves for Clonogenic Cells of a Murine Keratinising Squamous Carcinoma. *Int. J. Radiat. Biol.*, **12**, 535.

REINHOLD, H. S. (1967) *Stralingsgevoeligheid van Tumoren*. Thesis, Rotterdam.